# Lung Cancer Screening in California and Los Angeles County: A Landscape Analysis

**DOI:** 10.7759/cureus.111619

**Published:** 2026-06-27

**Authors:** Haley I Tupper, Yulei Angelina Gao, Amy L Cummings

**Affiliations:** 1 Surgery, University of California Los Angeles, Los Angeles, USA; 2 Neuroscience, University of California Los Angeles, Los Angeles, USA; 3 Hematology and Oncology, University of California Los Angeles, Los Angeles, USA

**Keywords:** california, healthcare policy, key stakeholders, los angeles county, lung cancer risk, lung cancer screening, medicaid, primary care, safety-net health systems, socioeconomic factors

## Abstract

Lung cancer screening with low-dose computed tomography (LDCT) reduces mortality, yet California has among the nation's lowest screening rates with substantial disparities by race, ethnicity, and socioeconomic status. This landscape analysis characterizes the current lung cancer screening implementation ecosystem in California and Los Angeles (LA) County to inform stakeholders seeking to improve screening equity.

We conducted a structured review of peer-reviewed literature and grey literature following methods adapted from Preferred Reporting Items for Systematic reviews and Meta-Analyses extension for Scoping Reviews (PRISMA-ScR) guidelines, synthesizing information on cancer screening policy frameworks, healthcare system structure, delivery infrastructure, risk factor distribution, and screening outcomes. The analysis examines lung cancer screening in the context of established screening programs (breast, colorectal, cervical cancer) and describes California's complex healthcare landscape with emphasis on safety-net systems serving the highest-risk populations.

Lung cancer screening currently lacks infrastructure elements present in established programs, including comprehensive federal legislative frameworks, quality regulation comparable to the Mammography Quality Standards Act (MQSA), and fully developed performance measures. California's healthcare system presents specific implementation challenges: MediCal reimbursement ranks 40th nationally with declining provider participation; multi-layered contractual arrangements create care coordination complexity; and federally qualified health centers face margin pressures while providing nearly half of MediCal primary care. LA County demonstrates profound geographic maldistribution-LDCT facilities cluster in low-risk areas while communities with the highest tobacco exposure and poorest air quality have the least access to screening resources. Tobacco retailers are concentrated in low-income areas, and three-quarters of those at-risk from poor air quality are people of color. Current performance reflects these barriers: California’s screening rates rank among the nation's worst, 68% of LA diagnoses are late-stage, and substantial racial disparities exist.

This landscape analysis provides stakeholders with comprehensive documentation of the policy frameworks, system structures, risk distributions, and implementation barriers that define the lung cancer screening ecosystem in the United States' most populous county, serving as a foundation for evidence-informed efforts to improve screening equity.

## Introduction and background

Lung cancer accounts for one in five cancer-related deaths [1] and screening with annual low-dose computed tomography (LDCT) can reduce cancer-specific mortality more efficiently than most other screening modalities [2,3]. Despite this effectiveness, screening uptake remains low nationwide [4] with California demonstrating particularly low rates among screen-eligible populations, defined as individuals ≥50 years old with ≥20 pack-years of smoking history within the past 15 years. This pattern is concerning because cancer risk is largely determined by environmental exposures accumulated over decades [5]. Carcinogenic exposures-including tobacco smoke, air pollution, and chronic inflammation-differ by education, income, and race due to longstanding sociopolitical reasons. The tobacco industry's well-documented history of targeting lower-income and predominantly Black neighborhoods exemplifies how environmental risks are unequally distributed [6,7].

As a relatively nascent screening modality, lung cancer screening can learn from well-established screening modalities, like mammography for breast cancer. Effective implementation requires understanding both the broader cancer screening landscape and the specific regional healthcare system that serves the highest-risk, historically neglected populations. However, stakeholders currently lack comprehensive documentation of how screening infrastructure and policies compare across cancer types, how California's complex healthcare delivery system creates implementation barriers, and how resources are distributed relative to risk. This landscape analysis reviewed both peer-reviewed journal articles and grey literature to help provide that foundation.

The first section examines the rationale for lung cancer screening, key regulations, and coverage determinations in the broader screening landscape, and models of care considerations. The second section describes California’s healthcare system with an emphasis on safety-net infrastructure and the distribution of lung cancer risk and management resources in Los Angeles (LA) County. Together, these sections provide an introduction to the current ecology of lung cancer screening in the United States’ most populous county. This analysis is intended for diverse audiences, including policy makers, health plans, health care delivery organizations, and advocacy groups, seeking to understand the landscape and pursue greater equity in lung cancer screening implementation.

## Review

Methods

This landscape analysis followed a structured approach adapted from Preferred Reporting Items for Systematic reviews and Meta-Analyses extension for Scoping Reviews (PRISMA-ScR) guidelines to characterize the lung cancer screening ecosystem in California and LA County.

Eligibility Criteria

Sources were eligible if they provided information on: (1) clinical effectiveness and policy frameworks for cancer screening; (2) federal or state legislation, regulations, or coverage determinations related to cancer screening; (3) California healthcare system structure, financing, and delivery; (4) LA County healthcare infrastructure and safety-net systems; (5) lung cancer risk factor distribution and screening outcomes in California/LA County; or (6) health information technology and care coordination relevant to screening implementation. No language, geographic (beyond primary focus), or date restrictions were applied.

Information Sources and Search Strategy

Searches were conducted in PubMed and Google Scholar (through December 2024) using terms related to: lung cancer screening, low-dose CT, cancer screening programs (breast, colorectal, cervical), healthcare delivery, MediCal/Medicaid, federally qualified health centers, and health disparities. Grey literature was systematically identified through targeted searches of: government websites [Centers for Medicare and Medicaid Services (CMS), California DHCS, California Department of Public Health (CDPH), Los Angeles County Department of Public Health (LACDPH)], legislative databases (federal and California bills/statutes), health system reports, insurance plan documents, and policy organization publications. Reference lists of key sources were reviewed to identify additional materials.

Selection of Sources

The primary author screened titles and abstracts for relevance, with full-text review of potentially eligible sources. Peer-reviewed literature was prioritized for clinical evidence, epidemiological data, and health services research. Grey literature provided essential documentation of current policies, regulations, payment structures, programmatic details, and local infrastructure characteristics not available in peer-reviewed publications. Historical sources were included when relevant to policy evolution; recent sources (2020-2024) were prioritized for current system characteristics and performance data.

Data Charting and Synthesis

Information was extracted and organized thematically according to the review's objectives: (Part I) lung cancer screening rationale, comparative cancer screening infrastructure, and care models; (Part II) California healthcare system structure and LA County risk distribution and management capacity. Data were synthesized narratively, with cross-referencing between sources to verify key facts and triangulate findings across complementary source types. No quantitative synthesis was intended or performed for this landscape review.

Results

Part I: The Lung Cancer Screening Landscape

The rationale for lung cancer screening: Lung cancer screening should be prioritized because it is highly efficacious, including compared to other screening modalities, and because it could particularly benefit certain socioeconomic groups who have been unequally exposed to lung cancer risk factors.

1. Clinical Impact and Mortality Reduction - Lung cancer is the leading cause of cancer-related mortality worldwide [8]. The landmark National Lung Screening Trial (NLST) established that annual LDCT screening of individuals with a significant tobacco use history achieved both enhanced cancer detection and a significant relative reduction in lung cancer mortality compared to conventional chest radiography. This study laid the groundwork for our current screening guidelines. Five-year survival differs dramatically by stage: 5.8% for Stage IV compared to 68.4% for Stage I (NSCLC) [9]. Screening with annual LDCT reduces lung cancer mortality by 20-26% [2,3], primarily through stage migration-enabling detection at earlier, more treatable stages. Annually, 1-3% of screeners are diagnosed with lung cancer, with the majority (50-70%) Stage I [10]. 

Tobacco exposure is estimated to cause 30% of all cancer deaths [11] and 85-90% of lung cancers [12]. Screening may also support tobacco cessation: NLST participants with abnormal screening results were significantly more likely to quit smoking and sustain abstinence compared to those with normal results [13].

2. Comparative Performance Across Cancer Screening Modalities - Despite its effectiveness, lung cancer screening uptake lags substantially behind other cancer screening modalities. LDCT is less invasive than colonoscopy, prevents death more efficiently than mammography or pap smear, and offers a relatively high reduction in cancer-specific mortality, yet lung cancer screening rates remain significantly lower than those for breast, cervical, or colorectal cancer (Table 1) [14,15].

**Table 1:** Cancer Screening Rates & Efficiency by Cancer Type ^a^: [14]; ^b^: [15]. BRFSS: Behavioral Risk Factor Surveillance System; gFOBT: guaiac fecal occult blood test; Flex Sig: flexible sigmoidoscopy.

Cancer	% of Eligible Screened in the United States (BRFSS estimates)^a^	Number Needed to Screen (to prevent 1 cancer-specific death)^b^
Lung	9.9%	130-320
Breast	70.2%	781
Cervical	77.7%	1140
Colorectal	66.9%	gFOBT: 1250 FlexSig: 864 Colonoscopy: 186

Use of standardized LDCT reporting improves diagnostic accuracy and simplifies the management of both incidental and non-incidental findings [16]. The Lung Imaging Reporting and Data System (LungRADS) reduced the false positive rate by more than 50% compared to the original NLST criteria [17].

3. Population-Level Risk Factors and Disparities - Substantial disparities in cancer outcomes already exist across racial, ethnic, and socioeconomic groups [18]. Without intentional implementation, lung cancer screening risks exacerbating these inequalities [19-21]. Tobacco use patterns reflect long-standing social determinants: only 5% of college-educated women smoke compared to 20-30% who have a high-school diploma or less, those living below the poverty line, or those insured by Medicaid [22].

This pattern creates a paradox in screening access. Although 27.8% of Medicaid patients use tobacco compared to 12.9% of privately-insured individuals [22], screening recommendations typically have the lowest penetration among Medicaid enrollees, the population at highest risk. Nearly one-third of people who use tobacco do not have a regular primary care provider (PCP), further limiting screening access [20]. The mortality consequences are stark: lung cancer mortality is five times higher among men with the least education compared to those with the most [23].

Although overall smoking rates have declined, tobacco industry practices continue to create unequal exposure. The tobacco industry has a well-documented history of targeted advertising in low-income areas, with tobacco retailers clustered near schools in these neighborhoods. A study of 30 US cities found nearly five times more tobacco retailers per square mile in the lowest-income neighborhoods compared to the highest-income neighborhoods [6,7]. Given this disproportionate risk burden, lung cancer screening resources should be concentrated in lower-income areas. However, the current screening landscape is regressive: those most at risk for cancer are least likely to be screened [24].

Cancer screening challenges and infrastructure at large: Lung cancer screening is nascent compared to other cancer screening. In 2013, the United States Preventive Services Task Force (USPSTF) recommended lung cancer screening based on NLST data, initiating broad insurance coverage: the Affordable Care Act (ACA) mandates private coverage of USPSTF A and B-level recommendations. A 2015 national coverage determination ensured Medicare coverage [25,26]. Medicaid, however, is not ensured or directly influenced by Medicare national coverage determinations (NCDs) and instead follows state-specific policies. Facing evidence that eligibility criteria excluded many at-risk African-Americans [27], USPSTF broadened the screening criteria to capture more at-risk individuals, followed by CMS modifying reimbursement requirements to improve access in 2022 [28,29]. The AAFP subsequently endorsed the USPSTF’s lung cancer screening recommendation in March 2022 [30].

In California, cost-sharing is prohibited for annual screening LDCT under Medicare, MediCal, and private insurance (except individual or grandfathered plans). However, prior authorization and plan-mandated screening locations remain barriers [31]. Screening access can be improved by reducing out-of-pocket costs and by minimizing mandatory touchpoints (chokepoints) for patients and PCPs. For breast, cervical, and colorectal cancer, financial barriers have been reduced, although not eliminated, through a combination of federal and state legislation, federal programs, and national and CMS coverage determinations.

1. Federal Legislation Regarding Financial Access - In 1990, Congress passed the Breast and Cervical Cancer Mortality Prevention Act (104 Stat. 409), directing the Centers for Disease Control and Prevention (CDC) to create the National Breast and Cervical Cancer Early Detection Program (NBCCEDP). The NBCCEDP provides screening, diagnostic work-up, and referral to treatment for uninsured or under-insured (screening not covered) women with annual incomes <185-250% (state-dependent) of the federal poverty line (FPL) [32]. In California, the NBCCEDP program operates as “Every Woman Counts,” and primarily serves uninsured women or MediCal limited-scope patients (e.g., pregnancy/emergency service only or unmet Share of Cost obligations) with an income <200% of the FPL [33]. Notably, Every Woman Counts requires PCPs to assess tobacco use in all enrollees and refer tobacco users to cessation programs.

In 1999, Congress passed the Breast and Cervical Cancer Treatment Program (BCCTP) Act [ House of Representatives (HR) Bill 106-468 Part 1], allowing states to provide Medicaid coverage for treatment of cancer through NBCCEDP. California implemented BCCTP in January 2002 for individuals <200% below the FPL requiring breast or cervical cancer treatment [34].

Colorectal cancer lacks equivalent comprehensive federal programs, although the CDC funded five “Colorectal Cancer Screening Demonstration Program” sites in 2004, partially modeled after the NCCEDP. In 2009, the CDC launched the Colorectal Cancer Control Program (CRCCP), which provides funding through a competitive application process to state or other organizations working with low-income populations to promote screening awareness and increase screening uptake [35].

2. CMS Coverage Determinations Regarding Financial Access - NCDs are guidelines developed by CMS to describe circumstances for Medicare coverage of a particular medical service and are binding for all Medicare contractors, health maintenance organizations (HMOs), competitive medical plans, and health prepayment plans. The Medicare Evidence Development & Coverage Advisory Committee (MEDCAC) advises CMS on NCD submissions. In the absence of an NCD, local coverage determinations (LCDs), representing 90% of Medicare policies, can be made at the discretion of a Medicare administrative contractor (MAC) [36].

3. Breast Cancer Screening Coverage - Several LCDs have reduced barriers to breast cancer screening and diagnostic follow-up. One LCD allows mammography coverage by self-referral, and permits mammographers to perform follow-up diagnostic imaging as needed after a screening mammogram without additional referrals, provisions in part made possible by the quality standards established through the Mammography Quality Standards Act (MQSA), detailed below. Specifically, screening mammography does not require a physician’s referral, provided the patient meets age criteria and has not undergone a prior screening mammogram within <11 months. However, facilities accepting self-referrals must have procedures for referring patients to qualified health providers in the setting of abnormal findings [37]. MQSA regulates both referral procedures and patient notification requirements.

Patients with a PCP can present for a screening mammography without a referral by providing their PCP’s name; if the provider does not accept the mammography report, then the patient is managed as a self-referral [37]. The same LCD eliminates written referral requirements for diagnostic mammograms following an abnormal screening, allowing patients to complete indicated diagnostic mammograms without additional provider touchpoints. However, while cost-sharing is prohibited for screening mammograms, it remains permitted for subsequent diagnostic work-up [38].

A separate NCD describes coverage of percutaneous breast biopsies using the Breast Imaging Reporting and Data System (BI-RADS) guidance. MQSA mandates BI-RADS assessment for mammograms, but only recommends it for ultrasound or MRI. Medicare covers percutaneous biopsy for BI-RADS III, IV, or IV, with image-guided biopsy permitted for non-palpable masses [39].

4. California State Legislation - California cancer-related legislation has had variable success in California under Governor Newsom. CA AB342 was approved in October 2021, eliminating cost-sharing for a colonoscopy following abnormal primary tests [e.g., fecal occult blood test (FOBT) or fecal immunochemical test- deoxyribonucleic acid (FIT-DNA)]. CMS subsequently removed cost-sharing for colonoscopies after abnormal primary tests for commercially insured (January 2022) and Medicare and Medicaid in expansion states (January 2023) [40]. State Bill (SB) 496 was also passed, requiring state-regulated plans, including MediCal, to cover comprehensive biomarker testing for clinical stage III/IV cancers. However, SB 257, which would have eliminated out-of-pocket costs for supplemental diagnostic breast imaging (ex. MRI, ultrasound, diagnostic mammogram), was vetoed in 2023 by the governor due to healthcare cost concerns. Reportedly, single-issue healthcare bills face difficulty passing unless they align with gubernatorial priorities.

5. Remaining Coverage & Financial Access Issues - Out-of-pocket costs for diagnostic work-up after abnormal screening results remain a significant barrier for breast, colorectal, and lung cancer screening. A federal bill (HR 5769) requiring health plans to cover diagnostic breast cancer imaging after screening without cost sharing has stalled since its introduction in 2021 in the absence of bipartisan support [41]. For lung cancer screening, average out-of-pocket costs for follow-up tests after LDCT were $424 in one study, with 7.4% of participants requiring a follow-up test after screening [42]. The National Lung Cancer Roundtable (NLCRT) Policy group is working to expand coverage and eliminate the cost burden for downstream work-up after screening LDCT.

Beyond financial barriers, operational complexities impede lung cancer screening implementation. Shared decision-making visits, which include eligibility determination and tobacco cessation counseling under a single billing code, must often be coordinated with LDCT scheduling. While not all insurances require prior authorization for screening LDCT, many do. Prior authorization processing time can prevent same-day shared decision making and screening, necessitating multiple patient appointments and creating barriers to screening completion [31]. Additionally, health plans may limit the number of covered counseling visits, which can further impact lung cancer screening [31].

6. Non-Financial Access Solutions - Standing orders and patient navigators both improve screening accessibility. Dedicated patient navigators, individuals who assist patients with navigating the complex health care system, have been repeatedly shown to significantly increase cancer screening rates for disadvantaged patients [43-46]. The Agency for Healthcare Research and Quality (AHRQ) and the CDC Community Preventive Services Taskforce strongly recommend standing orders to further increase the provision of preventive services, including mammography, based on evidence of improved screening rates [47-49]. Standing orders authorize designated medical staff (e.g., nurses, medical assistants) to order medical services following strict protocols without repeated physician authorization for each patient. Standing orders are particularly valuable in team-based primary care, redistributing workload and allowing providers to focus on acute or complex medical issues. Protocolized order sets can include conditional logic (e.g., last screen was normal) and can be integrated into electronic health records with automated care gap alerts [49]. The decision to use standing orders is largely institutional, informed by risk-management decisions. CMS permits standing orders when they are based on nationally-recognized evidence-based guidelines, although some insurance companies may not reimburse for services provided using standing orders [50]. Partnering with insurance plans can help reduce potential coverage barriers.

Notably, for lung cancer screening, the 2022 CMS NCD update removed practitioner requirements for the shared decision, which includes eligibility determination and smoking cessation counseling. Any qualified staff member can now provide shared decision making, provided all necessary components of the visit are appropriately documented) [25]. This change facilitates standing order implementation for lung cancer screening.

7. Legislation Regulating Quality - MQSA serves as the precedent for quality regulation in cancer screening. First passed in 1992 and subsequently amended, MQSA requires all mammography facilities except the Veterans Administration (VA) to obtain accreditation from an approved body and FDA certification to provide services. Only FDA-certified facilities are eligible for mammography reimbursement [51].

The MQSA governs a breadth of mammography standards, including image acquisition technology, radiology technologist and radiologist training requirements, standardized reporting in accessible language for both referring providers and patients, timely result transmission and notification, and standardized result transmission formats (including images). This regulatory framework establishes both quality baselines and operational consistency across mammography facilities [51].

Lung cancer screening lacks comparable quality regulation. The CMS NCD requires that lung cancer screening with LDCT “must be furnished in a radiology imaging facility that utilizes a standardized lung nodules identification, classification, and reporting system” [29] but does not mandate specific systems such as BI-RADS or LungRADS. This creates variability in reporting standards and quality benchmarks across lung cancer screening facilities.

8. Performance Measures - Performance measures are important for both data gathering and benchmarking, as well as driving clinical utilization. Among the most influential are Healthcare Effectiveness Data and Information Set (HEDIS) measures, developed and maintained by the National Committee for Quality Assurance (NCQA). HEDIS measures, which are revised and undergo a period of public comment annually, can be applied differentially to Medicaid, Medicare, and commercial insurance. For example, the colorectal cancer screening HEDIS measure existed in Medicare and commercial data sets for years but was only added to the core set of Medicaid adult health care quality measures in 2023 after significant advocacy.

HEDIS measures are typically collected at the health plan level, but directly affect federally-qualified health centers (FQHCs) because managed care plans use them to track patient care and tie quality reimbursement to performance. The Core Quality Measures Collaborative (CQMC), a public-private partnership between CMS and America’s Health Insurance Plans (AHIP), a trade association of health insurance plans, also uses many HEDIS measures as part of their core sets (e.g., Core Set for Accountable Care Organization (ACO)/Patient-Centered Medical Home/Primary Care) to drive assessment of quality and to inform value-based purchasing [52].

A HEDIS measure for lung cancer screening is currently in development with anticipated availability around 2026-27. Following the colorectal cancer screening experience, advocacy will be essential to ensure inclusion across all insurance datasets (Medicaid, Medicare, and Commercial) rather than selective implementation [53].

9. System Interoperability & Tracking Screening Results - Improving health information systems should be a priority for improving lung cancer outcomes.

a) Health information systems interoperability: A central challenge to high-quality screening is the lack of interoperability between health information systems (HISs), including electronic health records (EHRs), picture archiving and communication systems (PACS), and radiology information systems (RISs). While vertically integrated care delivery systems can more easily coordinate screening, diagnosis, and treatment, a large proportion of cancer screening is initiated in the community in non-integrated health centers.

Patients receiving care at hospital-affiliated health centers may access imaging-based cancer screening at their hospital-affiliated outpatient imaging centers (depending on insurance contracting), but many community health centers rely on independent diagnostic testing facilities (IDTFs) with discrete HISs. Without mandated intrinsic interoperability, provider organizations must either use manual labor to transfer health information or build individual application programming interfaces (APIs) between systems. Some community health centers (CHCs) rely on patients bringing in CDs and result reports, while some IDTFs offer cloud-based imaging portals where staff can individually download patient results.

When interoperability is built between HISs, the degree of interoperability can vary substantially. For example, an IDTF’s system may “push” results to the CHC’s EHR, but results still require manual release or movement into appropriate sections of the correct patient’s EHR. Resource-rich healthcare delivery organizations often build full interoperability, foundational, structural, semantic, and organizational between their HISs and those of priority partner organizations.

b) Tracking screening results: Tracking the percentage of eligible individuals who are screened can generally be extracted from most EHRs, but tracking screening results with appropriate follow-up presents greater challenges. Even with full integration of HISs across organizations, tracking both benign and abnormal screening results remains labor-intensive. Without full integration, CHCs must rely on manual entry of mammography results into registries and thus typically track only abnormal findings.

For breast cancer, specialized software facilitates mammography tracking, including automated patient and provider reminders. MagView, a comprehensive mammography information management system, automates risk-calculation, reporting, tracking, notification, recall, and quality assurance functions for breast imaging. Large systems, including the VA, University of California (UC) system, and Los Angeles County Department of Health Services (LACDHS), have adopted MagView to facilitate breast cancer screening quality management. While primarily targeted for and adopted by radiology, MagView reportedly has primary care tracking capabilities and offers a remote portal allowing diagnosing or treating providers to view external images. Cost is a reported adoption barrier.

MagView’s functionality relies on standardized language usage established through the BI-RADS Atlas, beyond standardized reporting structures alone. It automatically performs functions that many centralized lung cancer screening programs rely on a program coordinator to execute manually, except for tobacco cessation counseling and incidental findings management. Although MagView is developing an equivalent lung cancer screening, development is reportedly limited by the lack of a standardized reporting language comparable to the BI-RADS atlas (or equivalent LungRADS Atlas).

c) Tracking diagnosis to treatment to surveillance: Tracking diagnostic and treatment outcomes becomes further complicated when patients receive care outside of their primary system. Although cancer stage, pathology, and treatment must be reported to state and federal registries, no mandate requires treating institutions to transmit this information back to primary care or referring physicians. Similarly, additional diagnostic tests obtained by treating providers would not be routinely captured by systems like MagView, further complicating post-treatment surveillance.

d) Emerging technology solutions: Several companies are developing AI-powered solutions for lung cancer screening. RadNet, the largest national provider of outpatient imaging, launched DeepHealth in 2023. Riverain Technologies offers unique suppression technology (“Clear Visual Intelligence”) that facilitates lung nodules identification on CT and has been selected by the VA’s Lung Precision Oncology program for early lung cancer detection. Oatmeal Health is a newcomer that uses AI-powered clinical care coordination for lung cancer screening.

10. Unique Barriers for lung cancer screening - While examining lung cancer screening in the context of other cancer screening programs is instructive, lung cancer screening has unique challenges. Identifying an eligible individual requires accurate identification of current and former tobacco use, followed by pack-year calculations that must be regularly updated. Mandated shared decision-making visits and tobacco cessation interventions are additional time-intensive requirements. Because IDTFs may not perform therapeutic activities, tobacco cessation intervention must be delivered separately by physicians or other authorized caregivers [31,54].

The breadth of potential incidental findings on LDCT exceeds that of mammography, colonoscopy, or colposcopy, requiring more complex clinical management protocols. Unlike mammography, LDCT is unlikely to be performed in-house in the primary care setting, although mobile screening may provide a solution. Similarly, lung biopsy, such as transthoracic or endobronchial ultrasound (EBUS), requires hospital-based facilities due to their invasiveness, unlike breast biopsy, which can be performed in outpatient settings.

11. Centralized Versus Decentralized Lung Cancer Screening Models - There has been an enthusiastic international debate about the merits of centralized versus decentralized lung cancer screening, but the U.S. healthcare system is far more complex and fragmented than the UK’s National Health Service or many European systems in terms of both payment and delivery. While integrated systems like the VA and Kaiser (closed, integrated payer-provider) and some health and hospital systems with affiliated primary care clinics (open integrated delivery) can facilitate coordinated screening, many at-risk patients receive care in fragmented systems.

a) Centralized screening programs: In a centralized model, screening is vertically organized by disease. Once identified as screen-eligible, typically through EHR algorithms or their PCP, the patient enters a disease-specific screening program with dedicated personnel coordinating key screening functions across the continuum. Pre-screening processes include eligibility verification, shared decision-making, and tobacco cessation counseling. The program coordinated LDCT ordering and scheduling. Post-screening management encompasses result notification, follow-up scheduling, and additional work-up initiation.

Centralized programs are typically situated in large tertiary care institutions with dedicated CT scanners or operate dedicated mobile units. These programs often employ dedicated patient navigators and maintain close relationships with specialized lung cancer treatment teams to facilitate seamless care for abnormal findings.

b) Decentralized Screening Programs: In a decentralized model, the primary care provider assumes responsibility for most screening functions. Research demonstrates that centralized programs have superior annual adherence, yet most preventive care and screening currently occur in CHCs with insufficient resources for a fully centralized model [55]. Geographic and insurance access to organizations offering centralized lung cancer screening programs is limited, and promulgating centralized screening as the sole option may exacerbate existing screening disparities [55]. Thus, the decentralized model represents the current reality for most at-risk populations, despite its operational challenges and lower adherence outcomes.

c) Technology-enabled hybrid approaches: Much of what lung cancer screening programs do manually in terms of result tracking, coordination, and patient outreach could be automated electronically, and has been accomplished for other cancer screening (ex. MagView for mammography). With improved HIS system interoperability, clinical decision support software incorporating established screening and diagnosis algorithms, and clear referral pathways to multidisciplinary teams for abnormal findings, many centralized best practices could be brought to community health centers. This approach could improve preventive care coordination for patients more broadly while maintaining accessibility for the highest-risk populations.

12. Primary Care & Cancer Screening - Although specialists champion particular cancer screenings, screening is primarily initiated in primary care settings that are becoming increasingly overwhelmed. COVID-19 amplified existing primary care shortages [56]. Preventive care is often overshadowed by acute issues, especially in under-resourced settings where patients often present with greater illness complexity. Uninsured, newly-insured, and Medicaid patients frequently have more complex medical and social needs [57].

Lung cancer screening represents only one of many evidence-based screenings for older adults. A recent simulation revealed that PCPs would require 26.7 hours daily to deliver guideline-recommended care to the average American patient panel with 14.1 hours dedicated to preventive care alone [58]. The authors conclude that “if clinical guidelines do not consider the time opportunity cost of an intervention, the gap between guideline-based and clinical medicine will persist,” noting that disregard for these time demands exacerbates the primary care provider shortage in underserved areas [58].

This analysis highlights the need for dual approaches: short-term interventions to reduce proximal lung cancer screening barriers and longer-term collaborative redesign of screening processes to avoid overburdening primary care providers and patients. Myriad secondary prevention screenings compete for providers’ time and attention; lung cancer screening cannot be considered in isolation from this broader preventative care context.

Part II: California and LA County Healthcare System Structure and Risk Distribution

Healthcare in California: Healthcare oversight is divided between two departments within the California Health and Human Services Agency: CDPH and the California Department of Health Care Services (DHCS). Health insurance plans are regulated by the California Department of Insurance (CDI) or the California Department of Managed Health Care (DMHC), depending on the type of plan [HMO, preferred provider organization (PPO), point of service (POS), exclusive provider organization (EPO)] and the underwriter.

1. Insurance Structure & Payers - Kaiser Permanente is the dominant insurer for commercial (35% of individual, 37% of small group, and 54% of large group enrollment), individual marketplace (36% of “Covered California” enrollment), and Medicare Advantage (44% of enrollment) [59]. Kaiser consistently has the largest positive margins (6.9% in 2020), representing 31% of all health insurance revenue (in 2020), with Blue Shield representing 10%, Anthem 9%, HealthNet (Centene) 9%, UnitedHealth 7%, LA Care 4%, and all others representing 30% [59]. Most DMHC-regulated insurers have averaged positive margins of 4.1%, with LA Care as the notable exception at -1.6% in 2020 [59].

a) MediCal: California operates the nation’s largest Medicaid program by enrollment nationally, serving nearly one-third of Californians [60,61]. While 74% of Medicaid enrollees nationally are enrolled in managed care plans, 94.5% of Medicaid enrollees in California are enrolled in managed care plans, and only 5.5% are fee-for-service [62]. Managed care operates through capitated payments to contracted provider networks with varying degrees of patient choice.

MediCal accounts for almost two-thirds of net patient revenues in California’s public (DHS) hospitals and nearly 75% of net patient revenues for primary care clinics [60]. Although MediCal’s share of the state budget has been relatively constant 16% of the California general fund, federal matching contributions have financed most eligibility and enrollment expansions, with federal sources supplying 70% of total MediCal [60,63].

2. Insurance (Payer) & Provider (Delivery) Contracts - Understanding the landscape of healthcare payer and provider contracts, which are increasingly multilayered, is essential to pursuing pragmatic change. To improve equitable access to lung cancer screening and downstream care in California, a basic conceptualization of these relationships is necessary. 

a) Provider organization and contracting: Healthcare delivery in California is defined by multi-layered contractual arrangements, with the state ranking 42nd out of 51 for healthcare openness and access [64]. Physicians typically affiliate through medical groups (clinically and financially integrated with shared resources and centralized billing) or independent practice associations (IPAs), or virtual networks where practices remain independent. In medical groups (“group practices”), physicians are generally salaried employees or shareholders with performance incentives [65]. Despite California’s ban on corporate practice of medicine, hospital/health system ownership of physician practices grew substantially between 2011 and 2019 (primary care: 24% to 42%; specialists 25% to 52%) [65].

**Figure 1:**
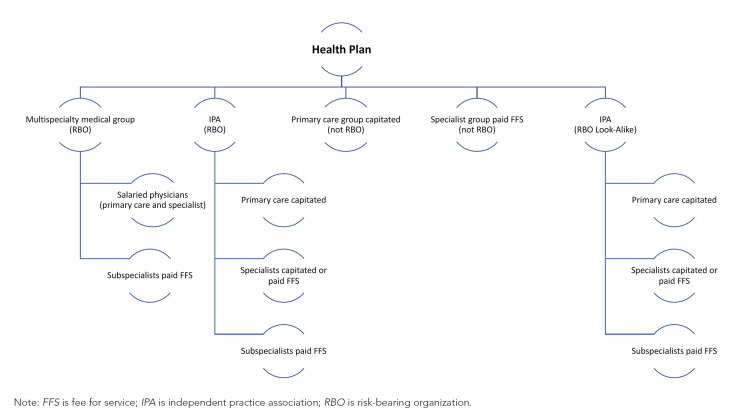
Sample Figure of Managed Care Contracting Arrangements and Delegation of Risk [65] Reproduced with permission from Jill Yegian.

Physician organizations often accept capitated payments with divided financial responsibility: professional services risk (outpatient care, diagnostics) versus institutional risk (inpatient services). Risk-bearing organizations (RBOs) and RBO Look-Alikes accept substantial MediCal professional risk from health plans [65]. Health plans commonly delegate credentialing, utilization management, care coordination, and network management to physician organizations, which contract with "downstream" providers and Management Services Organizations (MSOs) for administrative services (see Figure 1) [65]. Multiple capitation and delegation layers exist, with IPAs often assuming professional risk and sub-capitating FQHCs for primary care [59]. These complex contractual layers create multiple points where lung cancer screening coordination and specialist referrals can be impeded, particularly for MediCal patients served by FQHCs.

b) MediCal reimbursement: MediCal reimbursement represents a critical barrier to care access. California's rates rank 40th nationally, and provider participation has declined despite increasing enrollment [66-69]. Between 2013 and 2019, full-time equivalent providers per 100,000 MediCal enrollees decreased from 59 to 39 for primary care and from 91 to 63 for specialists [69]. Lower Medicaid reimbursement is also associated with an increase in emergency department visits for recipients [69]. Physicians report far greater difficulty obtaining imaging referrals for MediCal versus private insurance (27% vs. 7%) and specialist care (39% vs. 6%) [69]. California has 30% fewer radiology technologists than the national level per capita [70]. MediCal adults experience significantly more delayed care and specialist appointment difficulty than Medicaid beneficiaries in other states (48% vs. 36%) [67].

Beginning January 2024, MediCal increased provider rates for primary care, maternal care, and non-specialty mental health services to 87.5% of Medicare rates [71], but this does not address specialist and inpatient access, critical gaps for lung cancer screening implementation.

3. Healthcare Delivery - Healthcare delivery spans from outpatient to inpatient care. To pursue equitable access to lung cancer screening, diagnosis, staging and treatment, we must understand where Californians receive their care, particularly safety-net care, and some of the basic challenges facing these safety-net organizations.

a) Outpatient Care: Community health centers, particularly FQHCs, serve as medical homes for many MediCal and uninsured patients [57]. In 2019, nearly two-thirds of FQHC patients had MediCal insurance coverage [72], and community health centers provided 43.7% of MediCal primary care visits [73]. Private FQHCs have grown substantially, driven by federal grants and ACA funding opportunities [57], with patient volumes increasing 60% between 2013 and 2019 [72]. Although many county health systems operate FQHC-status clinics, many have downsized their primary care capacity [57].

FQHCs face significant workforce challenges, competing with high-resource systems like Kaiser for a shrinking primary care provider pool [57], with personnel expenses accounting for 70-75% of budgets [72]. Specialist care access is even more difficult. Community health center margins declined from 6.5% (2016) to 2.5% (2019), largely driven by increases in staffing costs [72]. Hospital affiliations improve FQHC’s ability to obtain diagnostic and follow-up care [57], but non-county hospital partnerships typically involve only a single hospital and 1-2 local FQHCs rather than broader networks [57]. EHR connectivity with non-FQHC providers or hospitals is “very limited," hindering specialist access and care coordination [57].

b) Inpatient Care: City/county provide 10% of all inpatient days but 15% of MediCal inpatient days, while non-profit hospitals account for 65% of all inpatient days but 57% of MediCal days. MediCal patients represent 65% of city/county hospital revenue compared to 25-30% at other hospital types [72]. City/county hospital operating margins, while improved between 2017 and 2019, remained negative in 2019 (-8.8%) [72]. Unlike other hospitals where patient revenue represents >94% of total revenue, city/county hospitals receive 75% of their total revenue from net patient revenues [72].

Although the UC hospitals and other private non-profit hospitals have disproportionate share hospitals (DSH) designation with safety net roles, these systems generally prioritize capacity for commercial patients with higher reimbursement. They support safety-net settings through direct funding, residents/staff, or in-kind contributions, allowing them to maintain tax-exempt status while costing less than directly treating MediCal patients [57].

**Figure 2:**
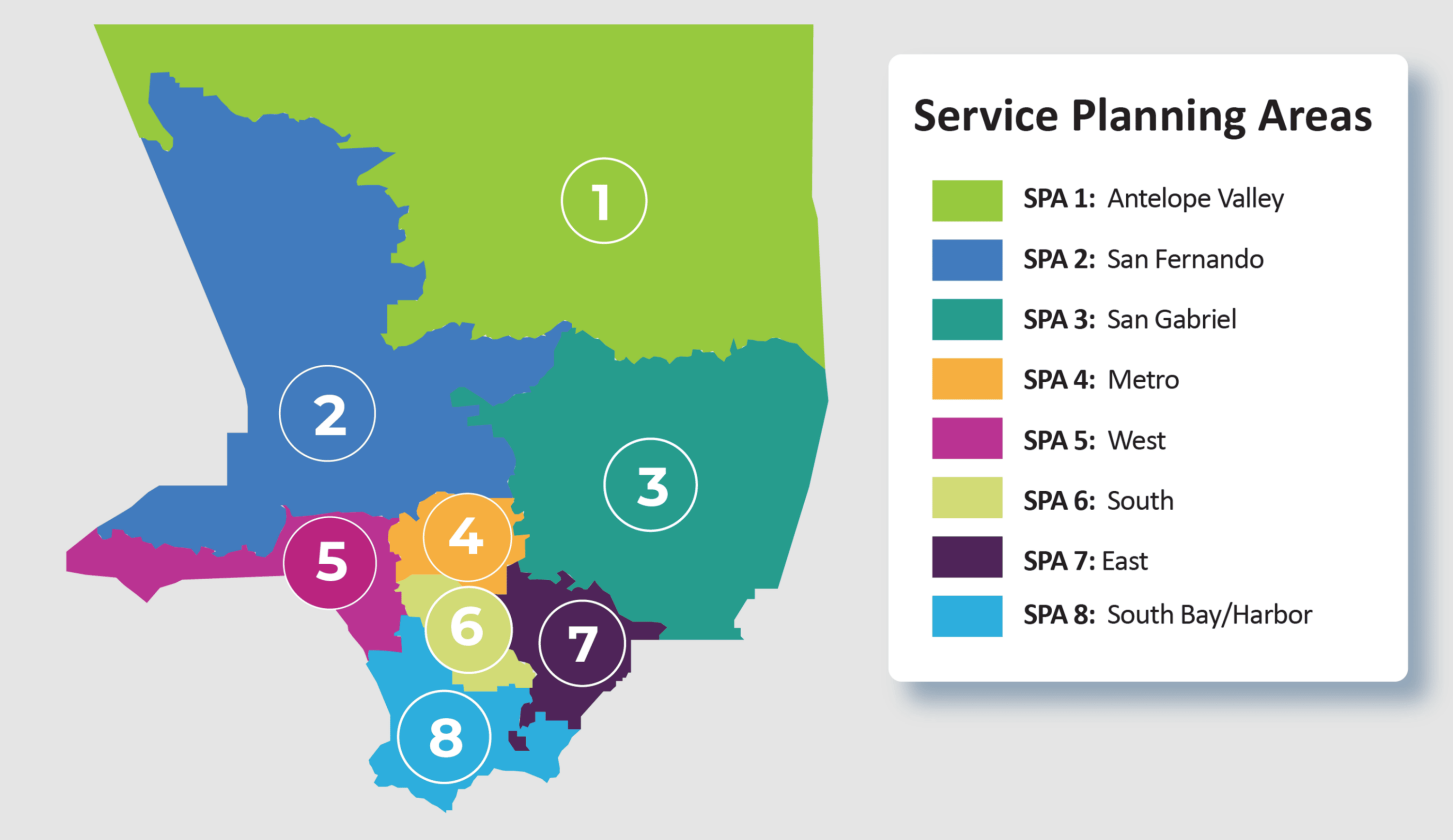
Map of LA County Service Planning Areas (SPAs) [76]. Reproduced with permission from Los Angeles County Department of Public Health (LACDPH).

Healthcare in LA County: With approximately 10 million residents, LA County has one-quarter of California’s population [74]. Compared to the statewide averages, LA county residents have lower incomes, less formal education, and higher unemployment, with one-third of residents living in households below 200% of the FPL, translating to higher MediCal enrollment [75]. LA County is divided into eight service planning areas (SPAs) with stark socioeconomic differences: 40.7% of SPA 6 (South LA) residents live below the FPL, compared to 9.5% of SPA 5 (West LA) (Figure 2) [75,76]. South LA has particularly high proportions of tobacco users, MediCal enrollees, and racial/ethnic minorities [77]. Limited public transportation and traffic contribute to regionally segregated health care delivery with significant workforce discrepancies: Despite having 160% of the West LA population, South LA has 90% fewer providers [75] with a deficit of over 1200 primary care providers [78].

1. Health Insurers: MediCal enrollment is sizable in LA and continues to grow. In 2012, 25% of the county was enrolled in MediCal, and in 2024, 42% was enrolled in MediCal [62]. As of January 2024, adults who met MediCal eligibility requirements could enroll, regardless of immigration status [79]. Despite this expansion, the rate of uninsured adults has remained steady around 7% with substantial post-COVID MediCal disenrollment occurring in 2024, largely due to paperwork challenges [80]. Approximately 36.9% of Medicare enrollees are dual-eligible for MediCare and MediCal [75].

LA County operates a “Two-Plan” MediCal Managed Care Model: publicly-run LA Care and commercial HealthNet (a subsidiary of Centene) manage care for approximately 4.1 million enrollees [62]. LA Care serves two-thirds of MediCal enrollees [75]. Both plans have full-risk agreements with partner plans [LA Care ® 1) Anthem, 2) Blue Shield; HealthNet ® 1) Molina]. LA Care, the largest insurer in LA County and the nation’s largest publicly owned health plan [63], retains risk for approximately half of its members, in addition to a shrinking pool of fee-for-service members [75,80]. In addition to the “Two Plan” model, Kaiser Permanente also serves some direct MediCal enrollees [81]. LA Care consistently has operated at negative margins (around -1%), but enrollment continues to grow, and it is the largest publicly-owned health plan [63].

 In addition to MediCal, approximately 5% of the LA population participates in Covered California (86% of enrollees are subsidized) with market shares as follows: Blue Shield (27.0%), Kaiser Permanente (25%), and LA Care (23%), with Health Net, Anthem Blue Cross, Oscar, Molina, and Sharp covering the remaining 25% [80].

2. Healthcare Delivery: Beyond Kaiser and the VA (integrated payer-provider systems), healthcare delivery in LA County is fragmented. LACDHS is technically vertically-integrated, but in reality, it is highly fragmented and sees far more patients in its emergency departments than its empaneled patient population [75]. MediCal (both LA Care and HealthNet) collectively pay global capitated payments for LACDHS for ~300,000 enrollees (~10% of MediCal enrollees), representing two-thirds of LACDHS’s 450,000 empaneled patients [75].

Other managed care patients receive outpatient care through capitated “participating physician groups” (PPGs)-provider networks including medical groups and IPAs-and inpatient care through fee-for-service (FFS) payments to contracted hospitals [75]. FQHCs typically maintain their own primary care medical groups, physician networks, and contract with IPAs and hospitals for specialist and inpatient care. Health Care LA, the country’s largest IPA, serves over 560,000 members and includes major FQHC systems (Watts, John Wesley Community Health (JWCH), Eisner, and St. John’s Well Child and Family Center) [57,82]. LA Care has been working to bypass PPGs and build a directly contracted physician network to reduce the number of contractual layers [75]. Notably, for delegated MediCal plan partners like Anthem, the MediCal PPG and hospital contracts have little overlap with their Medicare and commercial networks [75].

a) Primary care: Community health centers (CHCs), which are predominantly FQHCs or FQHC look-alikes (LALs) but also include free clinics and rural health centers (RHCs), provide care to nearly 20% of Angelenos [79]. FQHCs are an “entry point” into the system for 1.7 million Angelenos [83], providing primary care to 30% of MediCal recipients [73]. AltaMed is LA County’s largest FQHC system and among the nation’s largest. The Community Clinic Association of LA County (CCALAC) serves as an HRSA-funded Health Center Controlled Networks (HCCNs) supporting collaboration on clinical and operational practices and data sharing) [84].

b) Outpatient imaging (i.e., independent diagnostic testing facilities (IDTFs): Most outpatient imaging for CHCs is completed at IDTFs, which occasionally also manage excess for hospital systems, such as the LACDHS system. FQHCs establish contractual relationships with IDTFs for outpatient imaging. AltaMed, for example, has contractual relationships with United Medical Imaging (UMI) for mammography, both in-house at some AltaMed sites and at UMI imaging centers. When outpatient imaging is needed, utilization review teams identify IDTF based on contractual relationships, insurance coverage, and patient proximity.

LA County’s two largest IDTFs are UMI and RadNet. UMI, based in LA and Orange County, is committed to MediCal patients, and several multi-site FQHCs in SPA6 commonly refer patients for CT imaging to UMI sites. RadNet, founded in LA and now the nation’s largest outpatient imaging provider, has led lung cancer screening advances through AI technology and advocacy, including driving legislation in Maryland to mandate insurance coverage of diagnostic testing after abnormal breast and lung cancer screens [85] and partnering with the Florida Lung Health Coalition [86]. RadNet contracts with the LACDHS system to manage excess imaging demand and to assist with lung cancer screening.

c) Specialists care: Ensuring adequate specialist access for MediCal patients is challenging, with high specialist rates straining IPA and FQHCs budgets [75]. While LACDHS provides specialty care for its 450,000 empaneled patients (300,000 MediCal), the county is not responsible for MediCal enrollees assigned to other networks [75]. Health Care LA, the county’s largest IPA, manages the physician network for many FQHCs and recruits specialist care in conjunction with MediCal managed care plans (LA Care and Health Net) with variable success [75]. MediCal managed care plans are ultimately responsible for ensuring adequate specialist networks under DHCS contracts [75].

Los Angeles FQHCs use an eReferral or eConsult telemedicine system managed by LA Care Health Plan to triage specialist referrals. Specialist reviews cases online through a shared EHR and then advises the referring PCP on how to treat the patient or schedule consultations [57]. Many FQHCs use virtual consultations to help manage demand [75]. This eConsult system operates separately from the LA County health system with a specialist panel [57]. Several full-risk agreements exist between small-to-medium safety-net hospitals and health centers in their geographic service areas, coordinated through IPAs and MediCal MCPs [57].

d) Inpatient care: LA County has California’s least concentrated hospital market with significant competition and lower operating margins (3.9% vs. 4.4% for statewide) [75]. The six largest hospital systems accounted for nearly half of inpatient discharges in 2018 (Kaiser Foundation (11.3%), Cedars-Sinai (10.6%), Providence St. Joseph Health (9.2%), LACDHS (6.7%), Dignity Health (5.8%), PIH Health (4.4%) [75].

Disproportionate share hospitals (DSH) serve vastly different proportions of low-income and MediCal patients. UC hospitals provide the smallest proportion among California DSH hospitals [UCLA Ronald Reagan: 16% MediCal utilization rate (MUR), 16.5% low-income utilization rate (LIUR); UCLA Santa Monica: 11% MUR, 11.5% LIUR]. Conversely, LACDHS hospitals serve the highest proportions (Rancho: 100%, 100%; Olive View: MUR 95%, LIUR: 95.3%; Harbor-UCLA: 90%, 90.2%; LA General: 87%, 87.5%) [87]. Three hospital systems are affiliated with NCI-designated cancer centers: City of Hope Comprehensive Cancer Center, UCLA Jonsson Comprehensive Cancer Center, and USC Norris Cancer Center [87].

e) Integrated care delivery: LA County has three integrated delivery systems (VA, Kaiser, LACDHS), though Kaiser and the VA have limited MediCal roles as integrated payer-providers. LACDHS is a closed network (except Antelope Valley) that cares for 450,000 empaneled patients (300,000 MediCal managed care enrollees; 150,000 uninsured) through 23 outpatient centers [75]. LACDHS responsibilities include providing care to incarcerated individuals. My Health LA recipients (undocumented LAC adults with income <138% FPL) were transitioned to MediCal as of January 2024. Out-of-network care is very costly for the county system, which is financially responsible when empaneled MediCal enrollees use other hospitals or emergency departments [75].

Cancer in California and LA County: Cancer risk and resource availability in California and LA County are not equally distributed.

**Figure 3:**
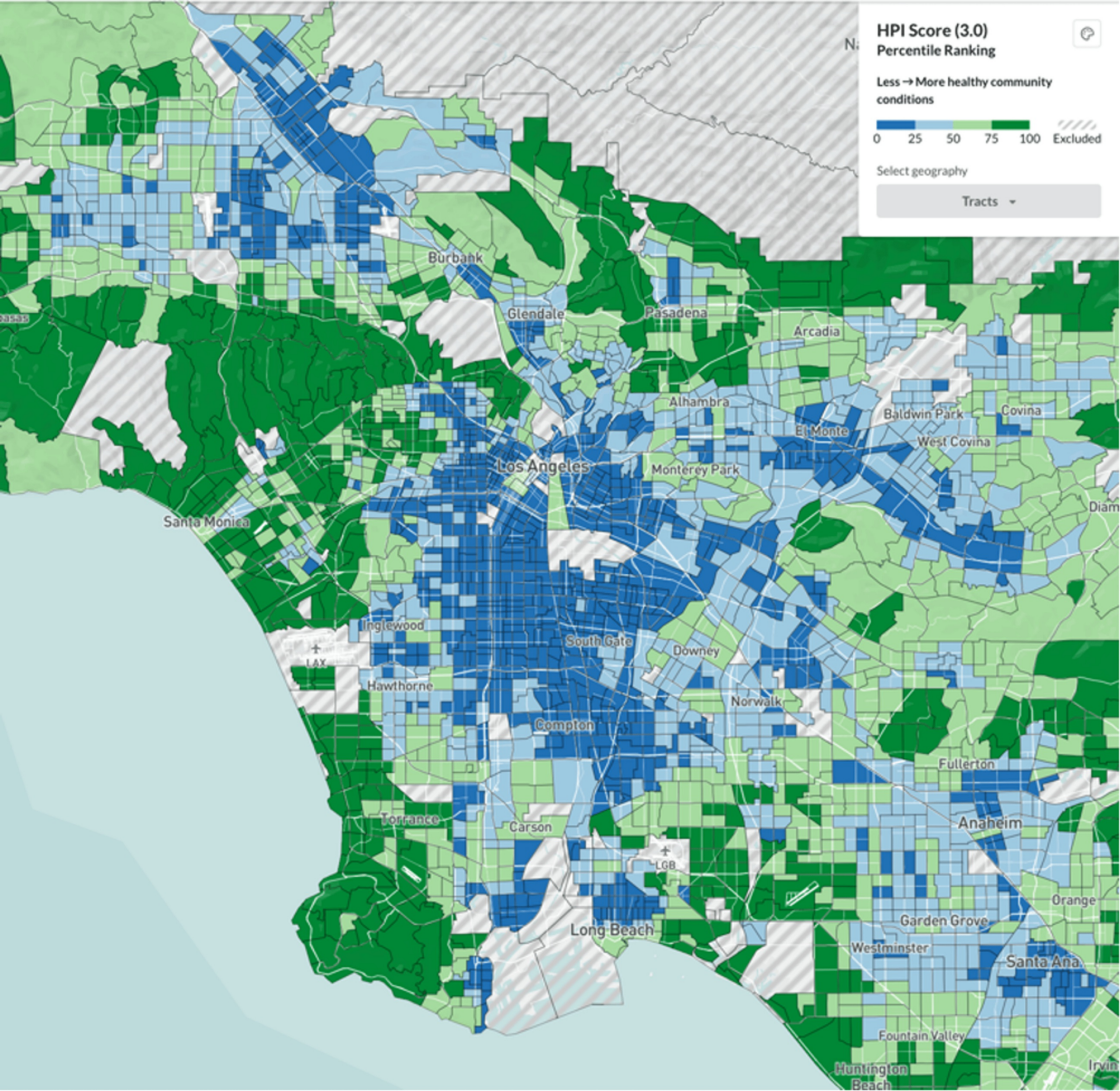
Healthy Places Index – Los Angeles County [91]. Reproduced with permission from Healthy Places Index.

1. General Cancer Screening - Adequate prioritization of preventive care, particularly cancer screening, remains challenging in California. The state ranks 43rd out of 51 for adults receiving age-appropriate screening, with rates declining from 71% to 65% (excluding lung) [88]. Systems with low (or negative) margins often prioritize diagnostic over preventative services unless screening is tied to additional financial support or incentives. Significant disparities exist in early detection, diagnosis, and treatment across all cancers in California [89].

2. Lung Cancer Exposure & Risk Factors in CA & LA County - Although California’s lung cancer incidence rate is below national averages, the state has the second highest number of new lung cancer cases (n=16,920) nationally, trailing only Florida, due to its large population [90]. Risk factors are inequitably distributed in LA County (Figure 3) [91]. 

a) Tobacco: Despite lower smoking prevalence than national averages (8.9% vs. 13.5%), there is a high absolute number of Californians and Angelenos at risk of lung cancer [92]. In LA County, 94% of residents live within a 10-minute walk of a tobacco retailer [7], and 29.6% of evaluated retailers engage in underage sales [93]. The strength of tobacco control policies and enforcement varies widely within LA County, with Santa Monica receiving an overall “A” tobacco control grade with 11 points and Whittier receiving an overall “F” tobacco control grade with 1 point (components: smokefree outdoor air, smokefree housing, reducing tobacco sales, and restrictions of flavored tobacco products) [94].

**Figure 4:**
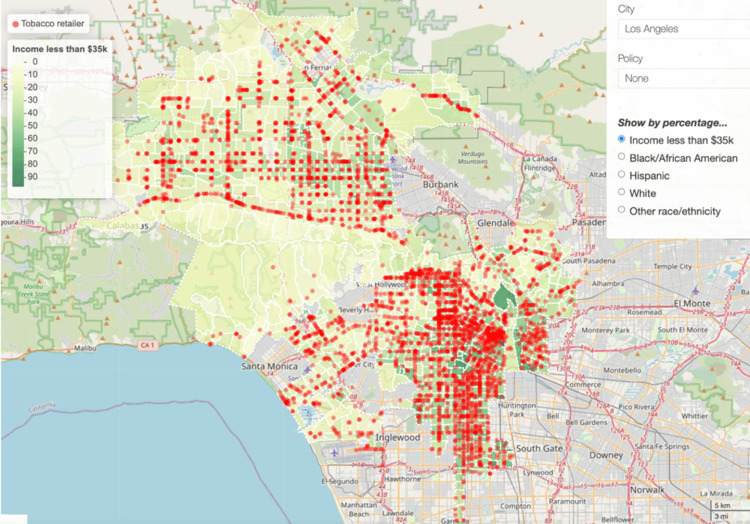
Tobacco Retailer Density & Household Income <$35k [7]. Reproduced with permission from the ASPiRE Center.

Smoking prevalence varies more than 4-fold within 81 incorporated cities in LA County [95], with high poverty zip codes showing elevated smoking rates [77]. Rates of 18-24% cluster around zip codes in South/South Central LA (SPA6), Downtown LA (SPA4), Huntington Park (SPA7), and the West LA VA area [77]. Tobacco retailer density demonstrates stark inequality: there are approximately 5 tobacco retailers per square mile in the highest income census tracts compared to 25 per square mile in the lowest income census tracts (Figure 4) [7].

The tobacco industry concentrates 90% of its marketing budget on consumer coupons, price discounts, and in-store advertisements, particularly in convenience stores [6], with targeted advertising in low-income and predominantly Black neighborhoods [96]. Flavored menthol cigarettes, often a gateway tobacco product, are $0.38 cheaper per pack in predominantly Black versus white neighborhoods with higher frequencies of price promotions and storefront advertisements [96].

**Figure 5:**
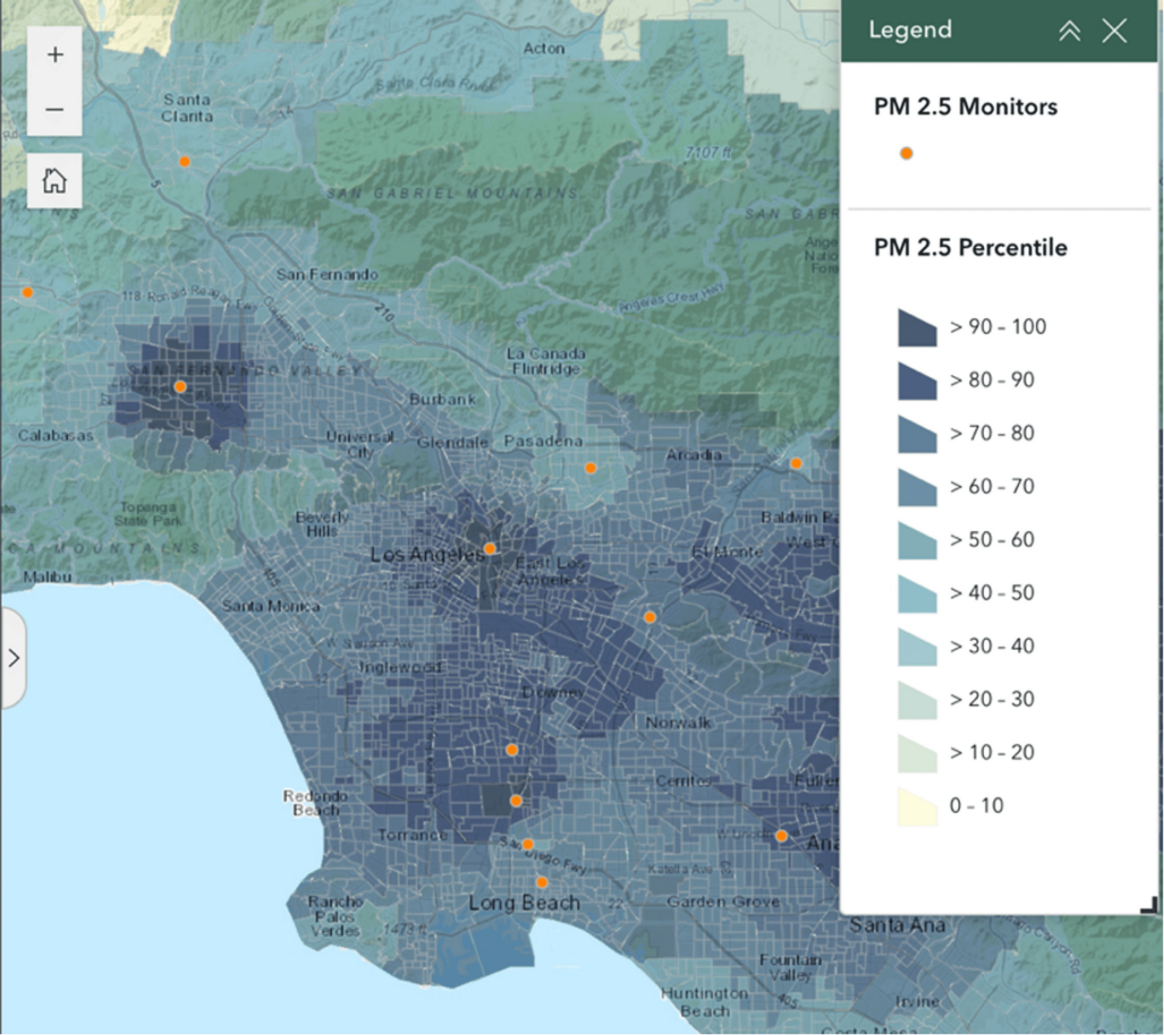
Particulate Matter 2.5 (PM2.5) in LA County [99]. Reproduced with permission from OEHHA. Map data sourced from the California Environmental Protection Agency. Office of Environmental Health Hazard Assessment.

b) Radon: Radon exposure is relatively low in LA County, with approximately 1% of homes exceeding EPA’s threshold [97].

c) Air pollution: The Los Angeles-Long Beach area ranks among the top 10 worst nationally for air quality among more than 200 metropolitan areas [98]. LA County received “F” ratings for both ozone and particle pollution in 2019-2021 [98]. However, exposure is unequally distributed: 74.7% of the 9.8 million Angelenos at-risk of developing health conditions from poor air quality are people of color, largely resulting from redlining, limited public investment, and deliberate placement of pollutants (power plants, industrial facilities, landfills, highways) in these neighborhoods (Figure 5) [94,99].

*3.* Lung Cancer Management in California & LA County - The lung cancer screening-to-diagnosis-to-treatment continuum is significantly impacted by the fragmented health insurance and delivery structures described above. Early detection only reduces mortality when followed by appropriate, timely treatment. Although California’s lung cancer mortality rate is below national averages relatively, the state has the second-highest absolute number of annual deaths (n=9320), reflecting both high incidence and failures in early diagnosis and treatment [90]. Significant disparities exist: Black Californians compared to White Californians experience lower rates of early-stage diagnosis (22.3% vs. 28.1%), surgical treatment (17.5% vs. 22.7%), any treatment (71.1% vs. 75.1%), and 5- year survival (24.9% vs. 29.4%) with similar patterns for Latino and indigenous Californians [92].

a) Screening: California has among the nation’s worst lung cancer screening rates, ranging from 0.7% to 13.9% of eligible individuals, depending on the data source [98,99]. Updated USPSTF criteria increased screen-eligibility by 81% [100]. California ranks 46th for lung cancer screening improvement rates [99]. Although Kaiser Permanente and the VA are excluded from these rates, anecdotally, lung cancer screening has not been a priority within Kaiser either. When performed, lung cancer screening is often low-quality: the ACR registry indicates 68.2% of California screening was appropriate compared to 86.7% nationally [101].

Low screening rates correlate with a low proportion of early-stage diagnoses: 25.9% of lung cancer cases are diagnosed early-stage in California, compared to 27.4% nationally, ranking California 36th among 47 states with data [92]. Information on early detection from incidental pulmonary nodules is limited in both LA and California.

**Figure 6:**
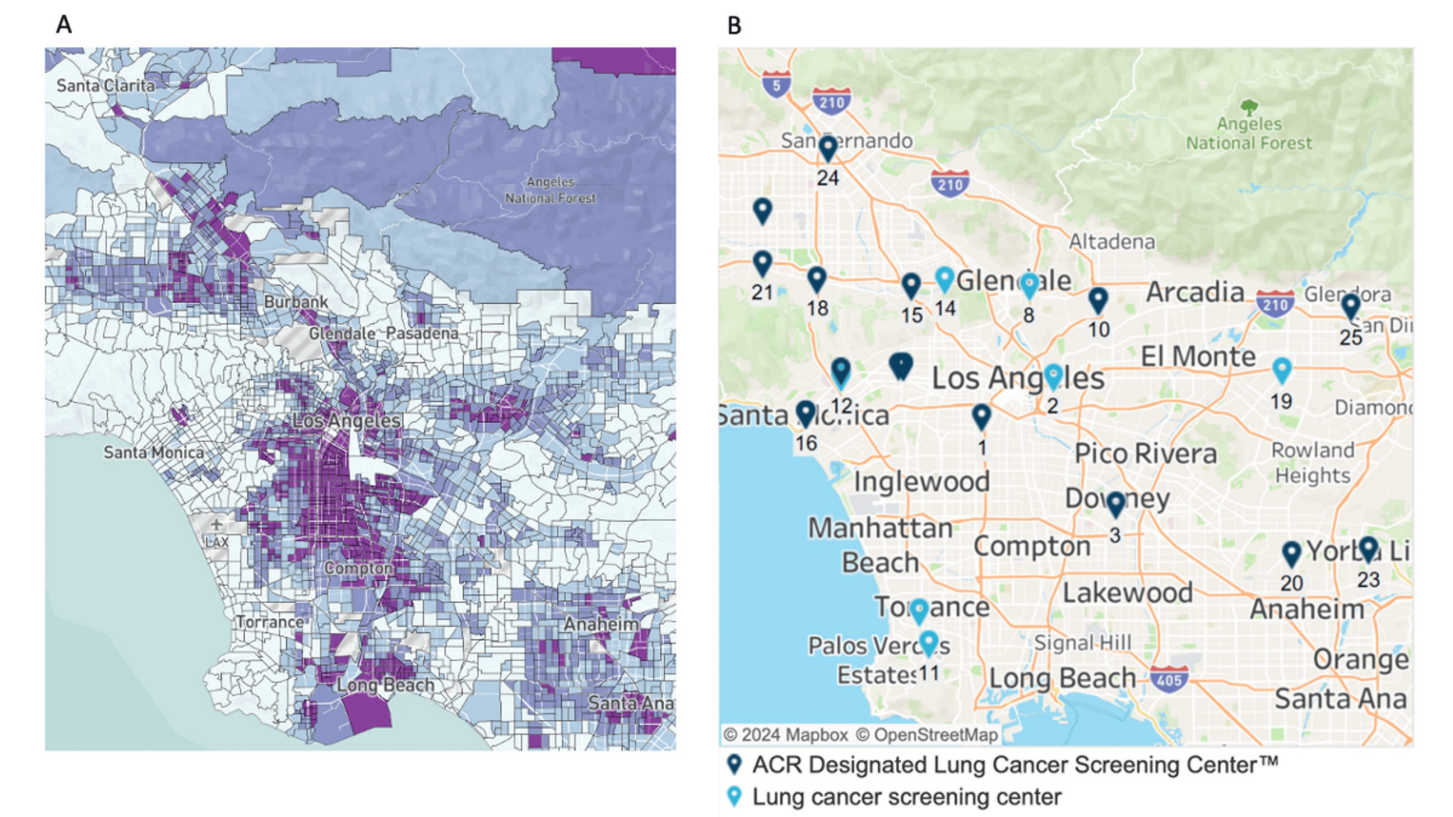
Geographic Maldistribution of Lung Cancer Screening Resources A: Percent of Adults Who Currently Smoke (Dark Purple: ~20% current smoking, Pale Blue: ~8% current smoking) [91]. Reproduced with permission from Healthy Places Index. B: American College of Radiology (ACR)-Registered Lung Cancer Screening Centers [102]. Map reproduced in agreement with the American College of Radiology (ACR) and the National Radiology Data Registry (NRDR).  The views expressed in this manuscript represent those of the authors, and do not necessarily represent the official views of the NRDR or the ACR.

CDPH’s 2021-2025 Cancer Control Plan aims to expand lung cancer screening in disadvantaged groups but lacks a clear implementation strategy or assessment of system-level needs [102]. In LA County, LDCT facilities and screening programs are clustered in relatively low-risk areas, making screening geographically inaccessible to areas with the highest tobacco use (Figure 6) [97,103]. Lung cancer screening rates correlate with CT scanner proximity [100,104]. Despite heightened risk in South/South Central LA, Downtown LA, and Huntington Park, these areas have the least access to screening resources.

b) Screening improvement initiatives: Several efforts have aimed to improve FQHC screening. Between 2015-2017, USC Keck partnered with Watts Healthcare to provide lung cancer screening at USC with dedicated navigator support, a community advisory board, transportation assistance, and a multidisciplinary committee for concerning findings (funded by: California Community Foundation; PI: Christopher Lee) [105]. The program ended when funding concluded in 2017. City of Hope has similarly worked to improve screening rates through provider education at two FQHCs [106].

Recently, the University of California health systems formed the UC Lung Cancer Coalition (UCLCC) to improve screening awareness and access. UCLCC uses Chorus, a cloud-based platform, to identify and pair potentially eligible individuals with care navigators through ucscreenca.org. However, UC systems serve variable proportions of low-income and MediCal patients.

c) Diagnosis & treatment: Late-stage diagnosis predominates: 68% of Los Angeles’ lung cancer diagnoses are late-stage [107] and 1 in 4 Californians receive no treatment [92]. While California’s surgical treatment rates are comparable to national rates, the state ranks 42nd out of 47 states for receipt of any treatment [92]. Improving equitable lung cancer screening requires establishing clear pathways diagnosis and treatment within local safety-net systems.

## Conclusions

Addressing socioeconomic disparities could eliminate more than one-third of premature cancer deaths, by some estimates, which would be far more effective than any individual cancer therapy. Lung cancer screening represents a critical opportunity to reduce cancer outcome inequities. Without intentional implementation, lung cancer screening risks further widening existing disparities by education, income, and race/ethnicity.

This landscape analysis documents the current state of lung cancer screening in California and LA County, revealing substantial gaps between risk distribution and resource allocation. The analysis examined lung cancer screening within the context of established cancer screening programs, identifying infrastructure elements-comprehensive federal legislation, quality regulation, performance measures, and technology solutions-that have facilitated implementation for breast and colorectal cancer but remain underdeveloped for lung cancer screening. The analysis also characterized California's healthcare system structure, including MediCal reimbursement challenges, multi-layered contractual arrangements creating care coordination complexity, and profound geographic maldistribution in LA County, where the highest-risk communities have the least access to screening resources.

Learning from established screening programs while addressing California's specific structural barriers-fragmented delivery systems, inadequate specialist reimbursement, and limited health information system interoperability-can inform more equitable implementation. This landscape analysis provides stakeholders, including policymakers, health plans, healthcare delivery organizations, FQHCs, and advocacy groups, with a foundational understanding of the policy frameworks, system structures, and implementation barriers that define the lung cancer screening ecosystem in the United States' most populous county. These insights can inform collaborative efforts to align screening resources with population risk, strengthen care coordination pathways, and ultimately reduce lung cancer mortality disparities.
